# Flap structure within receptor binding domain of SARS-CoV-2 spike periodically obstructs hACE2 Binding subdomain bearing similarities to HIV-1 protease flap

**DOI:** 10.1038/s41598-022-20656-z

**Published:** 2022-09-28

**Authors:** Michael H. Peters

**Affiliations:** Virginia Commonwealth University, Richmond, VA 23284 USA

**Keywords:** Biophysics, Microbiology, Diseases

## Abstract

The SARS-CoV-2 prefusion spike protein is characterized by a high degree of flexibility and temporal transformations associated with its multifunctional behavior. In this study, we have examined the dynamics of the Receptor Binding Domain (RBD) of the SARS-CoV-2 spike protein in detail. Its primary, binding subdomain with human Angiotensin Covering Enzyme II includes a highly conspicuous flap or loop that is part of a beta hairpin loop structural motif. Dynamic details of the RBD obtained through RMSF and Order Parameter calculations are consistent with structural details including the stability of “glue” points or dominant interaction energy residues of the RBD in the Up and Down states with its neighboring N-terminal domain (NTD) protomer. The RBD flap in the Up state protomer periodically obstructs the binding site on an approximate 70 ns time interval and is reminiscent of an HIV-1 protease polypeptide flap that opens and closes to modulate that enzymes activity. No claim is made here regarding the possible modulating role of the flap; however, the flap may be a potential site for therapeutic targeting aimed at keeping it in the closed state, as previously demonstrated in the inhibition of the HIV-1 protease polypeptide. The RBD primary binding subdomain is further shown to have not only similar dynamics but, also, an approximate 30% sequence similarity to the HIV-1 protease polypeptide.

## Introduction

Recently, we reported on ab initio dominant energetic mappings of SARS-CoV-2 trimeric spike protein including variants and lineages^1,2^. Dominant energetic mappings were shown to help predict structure–function changes in spike protein variants, including changes between so-called “Up” versus “Down” states of the receptor binding domain (RBD) of each protomer, where the RBD Up-state is more exposed and able to readily bind to human Angiotensin Converting Enzyme II (hACE2) on the surface of host epithelial cells. In our previous study^1^, we also performed dominant energetic mappings or “glue” points of the receptor binding domain (RBD) of the WT or Wuhan strain of spike with its predominant cell surface receptor protein hACE2 from its *static structure file* (PDB ID:6M17^3^). These mappings revealed the dominant amino acid residue binding pairs between the RBD and hACE2 that are responsible for the strong nanomolar binding properties experimentally observed for these two proteins^3–5^ (no attempt is made here to review the multitude of experimental binding studies for these two proteins). In general, the interaction energies are dominated by partial charge interactions of the residue constituent atoms or Coulombic potential interactions, as opposed to hydrophobic, van der Waals type interactions. In general, the RBD of the spike protein is highly flexible and undergoes fluctuations and dynamics that play key roles in the spike’s overall function and behavior. Therefore, dynamic simulations and analysis may play an increased role in assessing the multitude of functional aspects of the spike protein. In the current study we focus on the dynamic aspects of the RBD of the spike protein, including its fluctuations and temporal transformations. We quantify these dynamic changes that include a highly conspicuous flap near the primary hACE2 binding region of the RBD. It is shown that the flap is part of a beta hairpin loop structure that has hACE2 primary binding residues and nuanced dynamic features. The flap may have potential as a therapeutic target and bears dynamic and sequence similarity to the HIV-1 protease polypeptide flap, although it’s possible modulating role in SARS-CoV-2 binding is unknown and no claims are made here in that regard.

## Results

**Figure 1:**
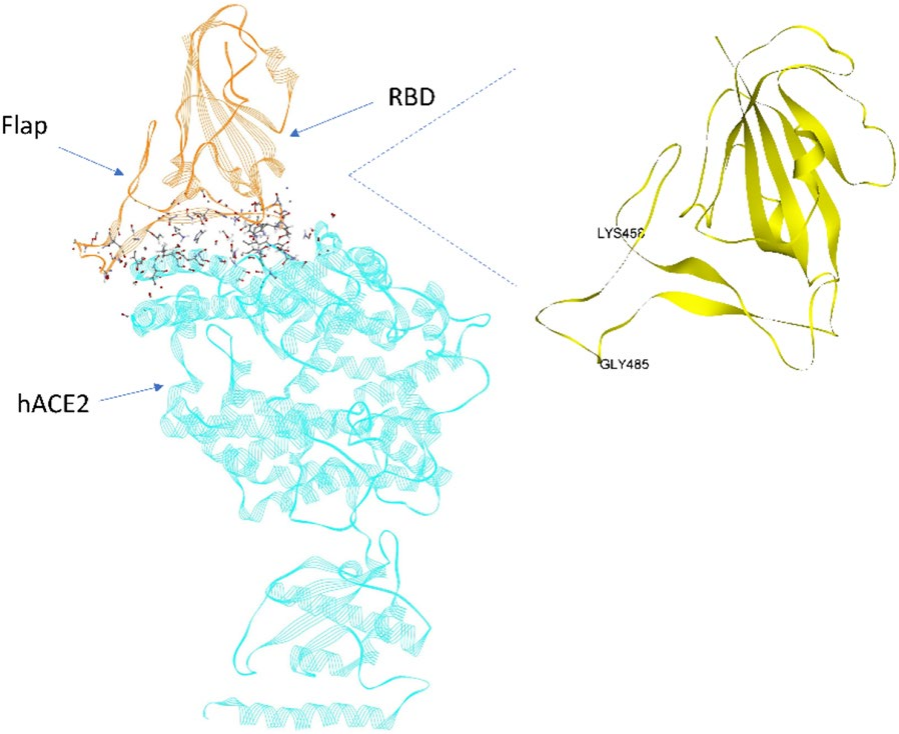
Dominant energetic atom–atom interactions (shown in sticks) between SARS-CoV-2 RBD and full length hACE2. A dynamic flap is also shown in this figure which opens and closes the dominant energetic binding subdomain of SARS-CoV-2 spike protein, as detailed herein.

**Figure 2:**
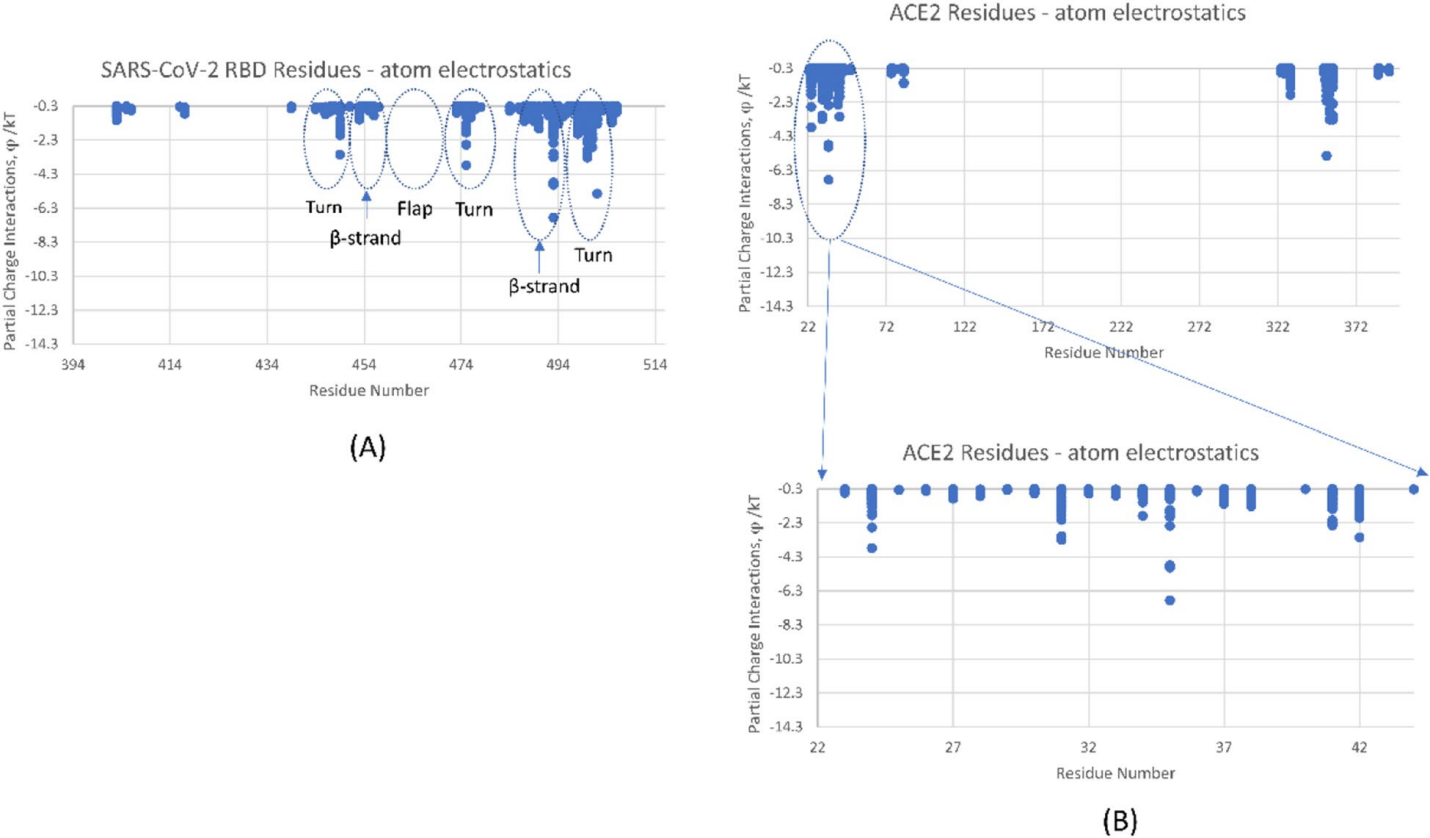
Partial atomic charge dominant interactions between SARS-CoV-2 RBD and hACE2 averaged over the last 10 nsec of a 200 nsec MD simulation. (**A**) RBD residues in the binding subdomain; (**B**) hACE2 residues with expanded view of its α-1 helix residues.

We first conducted 200 ns (nsec) MD simulations on the RBD-hACE2 structure file^3^ in order to verify the persistence of dominant energetic interactions over time (Figs. 1 and 2). These dominant interactions span residues S443 to N506 as shown more quantitatively in Fig. 2. This subdomain of the RBD consists of two centered, anti-parallel beta strands (L492-S494, L452-R454) flanked on one side by a highly flexible loop (L458-G485); together these structures are called a “beta hairpin loop” motif, which are ubiquitous across proteins, in general. The opposite flank with turns near residues T500-N501 and N439-N422 is stabilized by the distal beta strands, residues N394-I402 and Y508-E516. The loop itself has a (hACE2 binding) turn domain and a “flap” or non-binding region as shown in Fig. 2. All of these general secondary structures were shown to be preserved throughout the simulation period for both ligand-bound and unbound trimeric spike protein shown below.

**Figure 3:**
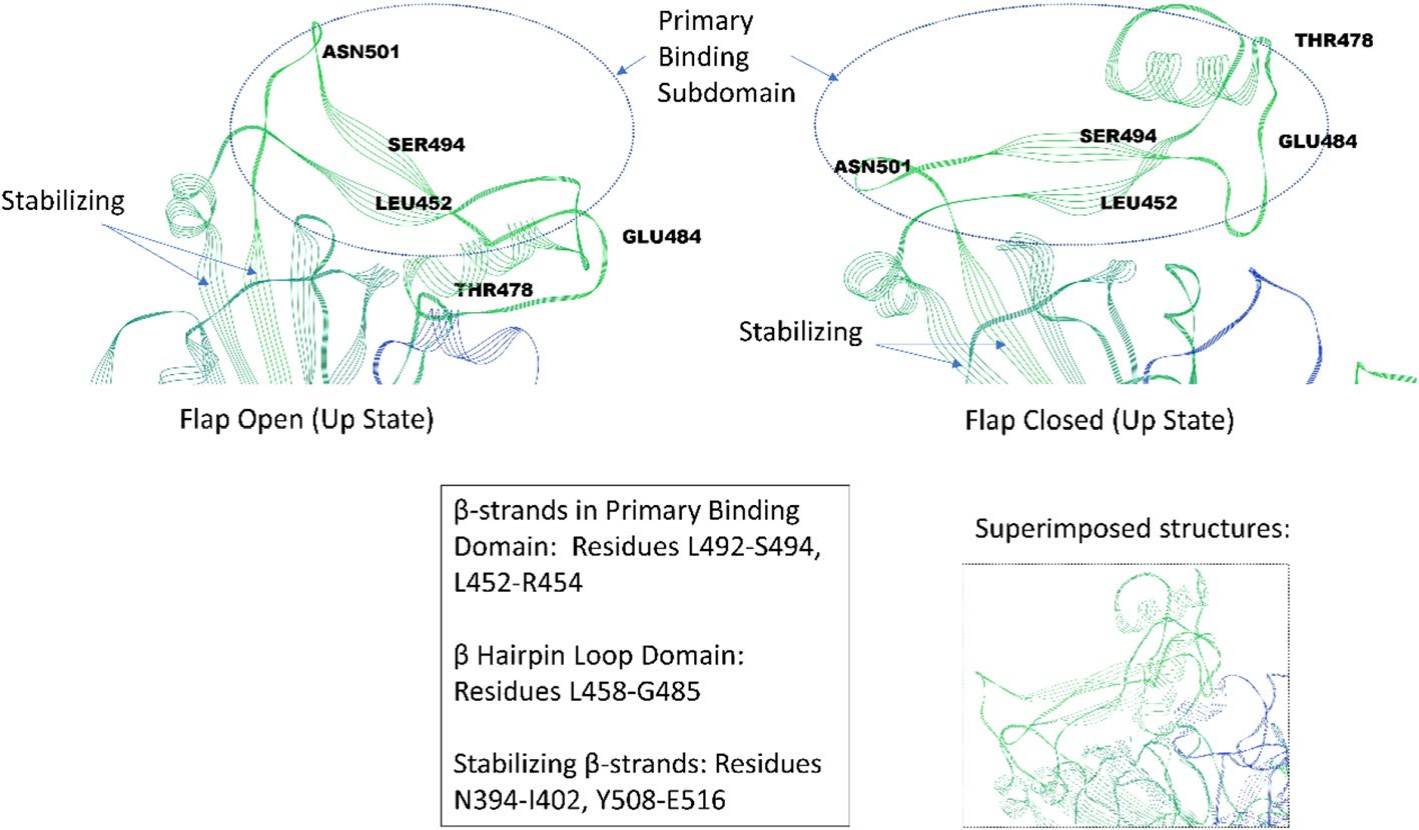
Flap open and closed states of the unbound, RBD subdomain region of SARS-CoV-2 trimeric spike for an “Up” state protomer (green). The adjacent “Down” state protomer is in blue and not fully shown.

Previously^2^, we also conducted molecular dynamics on the *unbound* WT trimeric spike protein (PDB ID: 6VSB^4^ and 6VYB^5^: one Up and two Down state protomers) where the simulations demonstrate that the flexible loop or flap for the Up-state protomer oscillates between a flap open and flap closed state several times over a 200 nsec period leading to an approximate flap dynamic time scale on the order of 70 nsec (Fig. 3; Supplementary Movie). This flap is seen to periodically obstruct the primary hACE2 binding site (closed state). Note the presence of a small helix (F464-E471) in the unbound trimeric spike protein in the loop region (Fig. 3) not present in the bound state (Fig. 1).

The highly conspicuous flap movement and periodic obstruction is also visible in other published MD simulations^6^ and consistent with Cryo-EM structure movie files of the trimeric spike protein^4^, although Cryo-EM movies cannot resolve exact movements in these highly flexible domains. The precise role of this flap, and if it plays any modulating role whatsoever, is certainly not clear, and no claim is made here regarding any such role. Our focus here is on the basic structure/dynamics of this flap for future considerations of its possible role in viral attachment, possible use in therapeutic efforts, etc. We note that flap dynamics for modulating binding in viral systems are known, for example, in the unliganded, HIV-1 protease polypeptide that also showed a similar beta hairpin loop or flap that opens and closes the binding site on the similar time scale of approximately 50 nsec^7^. Those flap dynamics have been shown to control ligand binding to the enzyme’s cleft^7,8^. It is to be noted that the Up-state subdomain binding region of the RBD, described above for hACE2 binding, is also readily identified from interprotomer dominant energetic mappings of the *unbound* trimeric spike protein (see Tables S1 and S2 of Ref.^1^). Specifically, residues V395-L518 in the Up-state protomer exhibit no dominant energetic interactions with its neighboring N-terminal Domain (NTD) protomer, as expected, whereas numerous residues in the same region of the Down-state protomer exhibited strong interactions with neighboring NTD protomer residues helping to keep this binding subdomain “hidden” and less available for hACE2 binding.

Despite the current unknown role, if any, of the flap or beta hairpin loop on spike protein function, therapeutics aimed at keeping a flap-closed state could represent a potential promising mitigation approach, as in the case of the HIV-1 protease therapeutics, and is of some warrant for additional quantification of its basic structure and dynamics, as given here. For example, a cyclic urea inhibitor helps keep a flap-closed state of the HIV-1 protease preventing ligand binding^7^.

**Figure 4:**
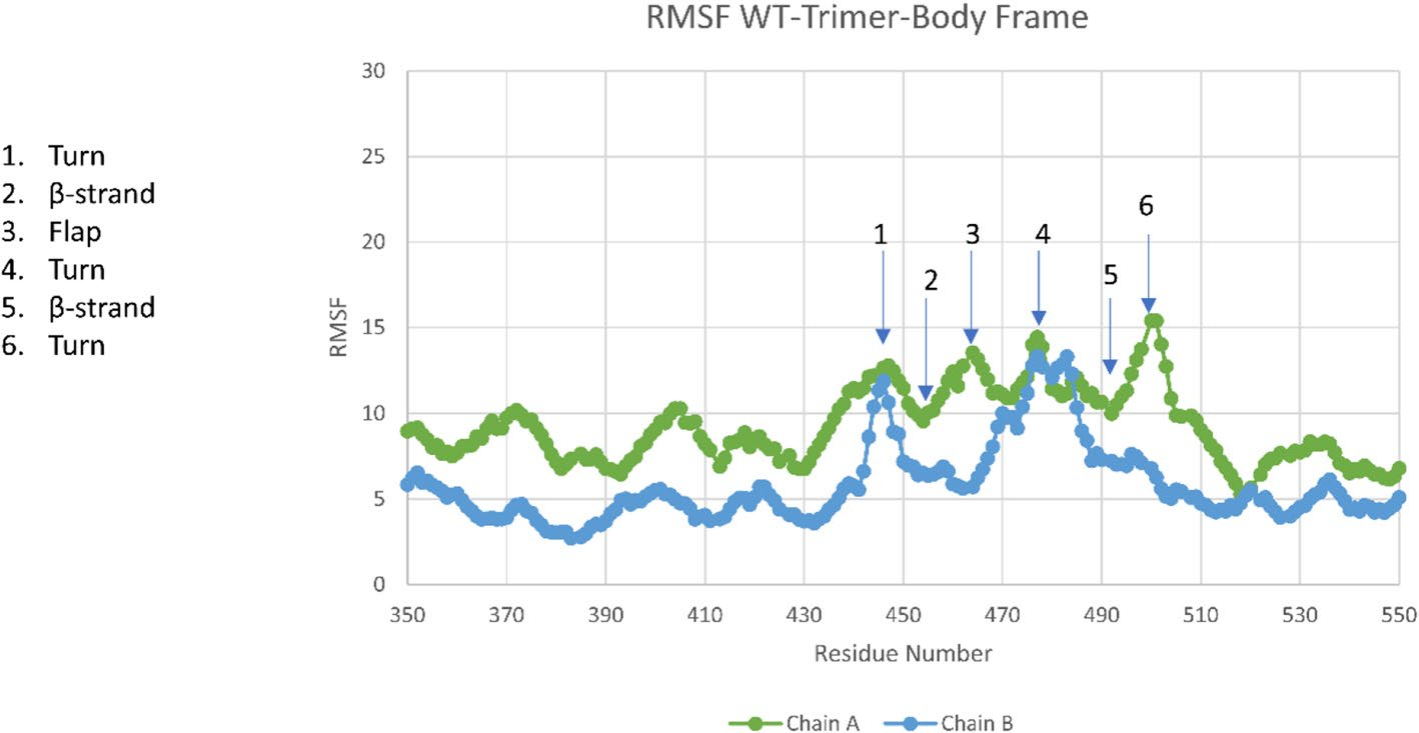
RMSF values for Up (Chain A) and Down (Chain B) chains of the WT Trimeric spike in the RBD binding subdomain. All expected translational motion features of this region are exhibited, viz., the inherent translational flexibility of turns and flap and the inherent stability of β strands in the primary binding domain (2,5).

Figure 4 shows predicted Root Mean Square fluctuations (RMSF) of the C-alpha residue atoms in the sub-domain binding region of the RBD corresponding to Fig. 3. The RMSF nicely captures all of the subdomain flexibility sites for Up and Down chains, as shown, including the flexibility of the flap and Turn 4, and the relative stability of β-strands (2 and 5). As expected, the flap and Turn 6 are relatively less mobile in the down state owing to interactions with its neighboring NTD (Tables S1 and S2 of Ref.^1^). Interestingly, Turns 1 and 4 are also mobile in the Down state owing to no observed dominant energetic interactions with its neighboring Down NTD protomer, as shown previously^1^. Also, of some interest, is the flap’s glue point residue ARG466 that forms strong interactions between the Down state RBD and the neighboring Down NTD^1^, and is clearly quantified in RMSF analysis (Fig. 4) over the entire simulation time. Repeat runs demonstrated consistency of these results, except for Turn 1 in the Down state owing to its high degree of flexibility (Supplemental Fig. S1).

Note that RMSF analysis does not capture orientational changes or the time dynamics of opening and closing of the flap. These dynamics require the analysis of time correlations or order parameters, as is done in assessing hinge motions of proteins or, in the case of HIV-1, protease flap dynamic analysis^7^. In passing, is also to be noted that order parameters are directly measured experimentally via depolarization anisotropy or H-NMR^9^, although this has not yet been determined for the spike protein to our knowledge.

**Figure 5:**
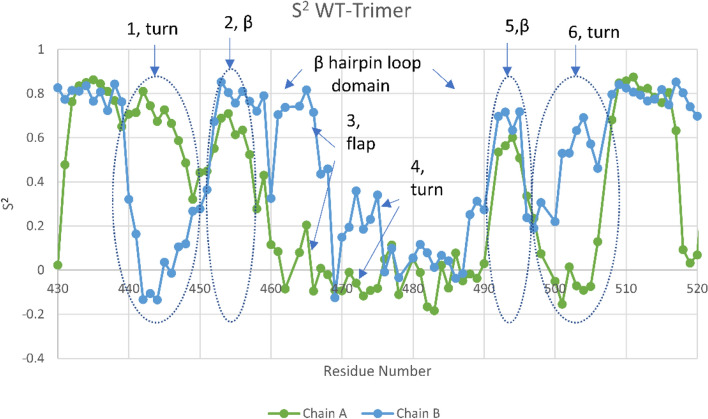
Order parameter (S^2^) for the primary RBD subdomain of SARS-CoV-2 (Wuhan-WT). Note that the Up state flap demonstrates a partial negative correlation associated with flap orientational changes on the average of approximately 90 degrees over 70 nsec periods.

Figure 5 shows the order parameters obtained for the RBD binding subdomain corresponding to the RMSF data of Fig. 4. Here we take the correlation function plateau values after a correlation Δt of 70 nsec; example of the correlation function behavior and the complete listing of values are given in the Supplemental Information (Fig. S2 and Table S1). Values of the order parameter near 1 are indicative of more rigid, local behavior, where the NH residue vector orientations do not change significantly; whereas smaller values near zero are indicative of more rotational “randomness”. Negative values can occur and are associated with negative correlations or NH vectors that on the average are approximately 90 degrees out of phase with an initial value. The hairpin loop domain demonstrates some negative correlations of the flap in this 70 nsec interval in the Up state, but not in the Down state, consistent with movies of the flap dynamics. All features are consistent with RMSF analysis including, for example, the role of flap residue ARG466 in stabilizing the Down state protomer. We repeated these simulations several times with consistent order parameter results, as given in the Supplementary Information (Fig. S2); as with the RMSF results, Turn 1 orientational behavior was the only region not consistent between repeat runs owing to its high degree of flexibility.

### Sequence homology

**Figure 6:**
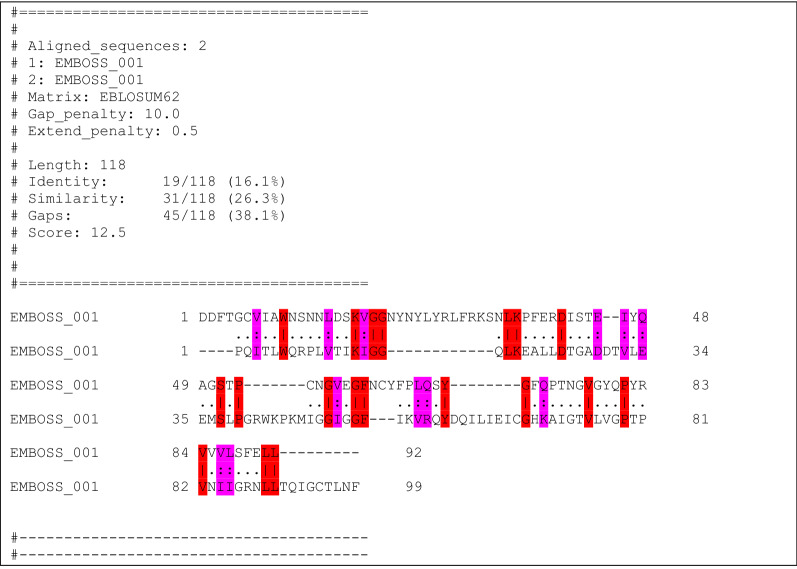
Pair structure sequence alignment^10^: RBD primary binding domain (residues D389-L518 shown; a complete RBD mapping is shown in Table S3) and HIV-1 protease polypeptide (PDB: 1HHP). Red highlights are identical residues and magenta highlights are similar residues. Identity, similarity and gap quantitative scores are reported above and can be compared to negative control (human beta hairpin loop Pin1; Table S2).

Because of the similarity with HIV-1 protease polypeptide flap dynamics, we also conducted sequence alignment tests between the SARS-CoV-2 RBD and the HIV-1 protease (PDB ID: 1HHP), as shown in Fig. 6. Remarkably, there was significant sequence homology found between the RBD binding and flap segment (V395-L518) and the HIV-1 protease. We also conducted a negative control with a non-virial beta hairpin loop structure (PDB ID: 2M8I) that demonstrated little homology with SARS-CoV-2 RBD (Supplemental Information, Table S2).

## Discussion

The spike proteins of the family *coronaviridae* have complicated or nuanced structure and dynamics that have evolved over centuries^11,12^. Here we have looked more closely at the dynamic features of the RBD of the Wuhan (WT) SARS-CoV-2 spike protein (genus *betacoronavirus*; subgenus *sarbecovirus*) in both its bound state with hACE2 and unbound state using ab initio, all-atom molecular dynamics. The RBD has a flap structure that is not involved in the primary binding hACE2 subdomain. The flap (loop) itself is part of a beta hairpin loop structure that contains turns and beta strands that *are* associated with the primary hACE2 binding residues of the RBD. It is to be noted that beta hairpin loop structures are ubiquitous in protein structure and function, in general. The flap is shown to oscillate between a binding site obstruction orientation (closed) and unobstructed state (open) on an approximate 70 nsec time period. To be clear, no claim is made here regarding the possible modulating role of the flap in SARS-CoV-2 spike protein behavior, and only its dynamics have been analyzed. Nonetheless, flap dynamics for HIV-1 protease have been previously studied and demonstrated to play a modulating role in binding behavior on a 50 nsec time scale, and inhibitors have been developed to help maintain a closed state preventing enzyme function. Significant sequence homology was also demonstrated between the primary binding subdomain of the RBD and HIV-1 protease, but further studies are needed to examine this relationship more fully. In addition, by studying differences between the Down and Up state interprotomer glue points, we have identified key NTD residues that help keep the RBD in the Down state and prevent any flap movement. These results may help guide the development of therapeutics, perhaps along the lines of the cyclic urea inhibitor of the HIV-1 protease, aimed at keeping the flap closed regardless of any modulating role. In addition, it is of further interest to examine what role, if any, mutations in the spike protein associated with SARS-CoV-2 variants play in the dynamic aspects of the beta hairpin loop structure, in general. Previously, we examined the dominant energetic mappings or glue points between the Up and Down state protomers across the genera of *coronaviridae*. By comparing those results with sequence alignment studies, we showed that many of these critical glue points were conserved across the genera, and it is of interest to examine the glue points and dynamics identified here associated with the beta hairpin loop across these genera as well.

## Methods

### Molecular dynamics

Explicit solvent molecular dynamics (MD) simulations were performed using the NAMD2 program^13^. We used the CHARMM36m force field along with TIP3P water molecules to explicitly solvate the proteins^14^. Simulations were performed maintaining the number of simulated particles, pressure and temperature (the NPT ensemble) constant with the Langevin piston method specifically used to maintain a constant pressure of 1 atm. We employed periodic boundary conditions for a water box simulation volume as well as the particle mesh Ewald (PME) method with a 20 Å cutoff distance between the simulated protein and water box edge. The integration time step was 2 femtoseconds with the total simulation time being approximately 200 ns for each protein system analyzed. All our protein simulations were conducted under physiological conditions (37 °C, pH of 7.4, physiological ionic strength). Our studies use all-atom molecular dynamic simulations of the entire trimeric spike protein over a 200 nsec period, which was shown to be sufficient in capturing domain motions of the SARS-CoV-2 spike protein^1^.

### RMSF and order parameter

Root mean square fluctuation data was obtained for C-alpha atom positions over a period of 200 ns. We discarded the first 50 nsec equilibration period of the 200 nsec total simulation time, which was previously determined as the approximate time required to reach a dynamic structural equilibrium from the initial *.pdb structure file^1^.

More precisely, the RMSF is determined from

$$RMSF=\sqrt{\frac{1}{N}{\sum }_{k=1}^{N}(\Delta {x}_{k}^{2}+\Delta {y}_{k}^{2}+ \Delta {z}_{k}^{2})}$$

where $\Delta {x}_{k}={x}_{k}$ − $\underline{x}$,$\Delta {y}_{k}={y}_{k} - \underline{y}$, etc. with ${x}_{k}$ denoting the x-position of a specific C_α_ atom for each kth time and $\underline{x}$ the average x position over the time ensemble, etc. (N is the total time ensemble number; 150 snapshots). As before^1^, because of overall protein drift in the lab frame, all x positions for the spike protein are measured relative to the body frame using VAL991 as the reference point in the approximately rigid S2 domain of the spike protein.

Order parameters were calculated for N–H bond vector rotational correlations for each residue based on 150 total “snapshots” over the simulation period. Again, data for the first 50 nsec were discarded. More precisely, the order parameter, S^2^, is calculated from the plateau values of the rotational correlation function

$$C(\Delta t)= {{<}{P}_{2}(cos \theta ){>} }_{\Delta t}$$

where ${P}_{2}$ is the second order Legendre polynomial, $\mathrm{cos}\theta ={\varvec{n}}\left(t+\Delta t\right)\cdot {\varvec{n}}\left(t\right)$, and ${\varvec{n}}(t)$ is a unit vector along the NH bond vector at any time t; <  > denotes an ensemble average for each time interval Δt. For rigid proteins, the order parameter would be one. For uniformly random orientations between π/2 and − π/2 the order parameter would be zero.

### All-atom energetic mappings

Previously^15^, we analyzed the complete inter-protomer and intra-protomer interactions across two independently published structure files (PDB ID: 6VSB and 6VYB; one up and two down protomers) for SARS-CoV-2 trimeric Spike protein using the open source energy mapping algorithm developed by Krall et al.^16^. This spatial and energetic mapping algorithm efficiently parses the strongest or most dominant non-covalent atom–atom interactions (charge and partial atomic charge, Born, and van der Waals forces), according to empirically established parsing criteria, based on the ab initio AMBER03 force field model. It allowed us to establish differences between Up–Down and Down–Down protomer interactions, which for example identified D614 as a key intra protomer interaction prior to the appearance of the alpha variant. Following our previous studies, the parsing criteria is taken as the upper limit of − 0.1 kT units for Lennard–Jones (van der Waals) criteria and − 0.3 kT units for Coulombic interactions, although lower values can also be specified in the analysis part of the mappings in order to further refine the results^16^. Note that in the all-atom analysis dominant van der Waals interaction forces are commonly associated with nonpolar atom–atom interactions and hydrophobic protein interaction regions, whereas the Coulombic partial charge and charge interactions are commonly associated with hydrophilic protein interaction regions and can include hydrogen bonding and backbone atom partial charge interactions.

### Sequence alignment

Pair structure sequence alignment is preformed using EMBOSS Needle^10^, which reads two input sequences and determines their optimal global sequence alignment including gaps. This alignment method uses the Needleman–Wunsch alignment algorithm^10^. Sequences were obtained directly from the PDB files: RBD (6M17)^3^, HIV-1 protease (1HHP)^17^, and nonsense control beta hairpin loop (2M81)^18^.

## Supplementary Information

Supplementary Figure S1.

Supplementary Figure S2.

Supplementary Figure S3.

Supplementary Table S1.

Supplementary Table S2.

Supplementary Table S3.

Supplementary Information 1.

Supplementary Video 1.

## Data Availability

The data sets generated and/or analyzed during the current study are available at: https://datadryad.org/stash/share/b4lqDIC6hlpxNCaHTNSNsrs4ArVQpJA0N-ijR8u2JWY.
