# Supplementary figures and images for: Flap structure within receptor binding domain of SARS-CoV-2 spike periodically obstructs hACE2 Binding subdomain bearing similarities to HIV-1 protease flap
Source: Sci Rep. 2022 Sep 28;12:16236. doi: 10.1038/s41598-022-20656-z (PMC9517965; doi:10.1038/s41598-022-20656-z)

### Repeat A

RMSF WT-Trimer-Body Frame

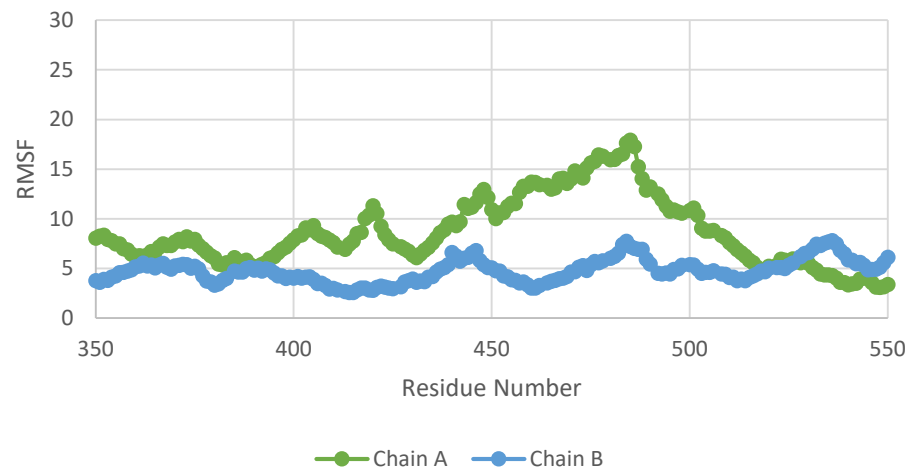

### Repeat B

RMSF WT-Trimer-Body Frame

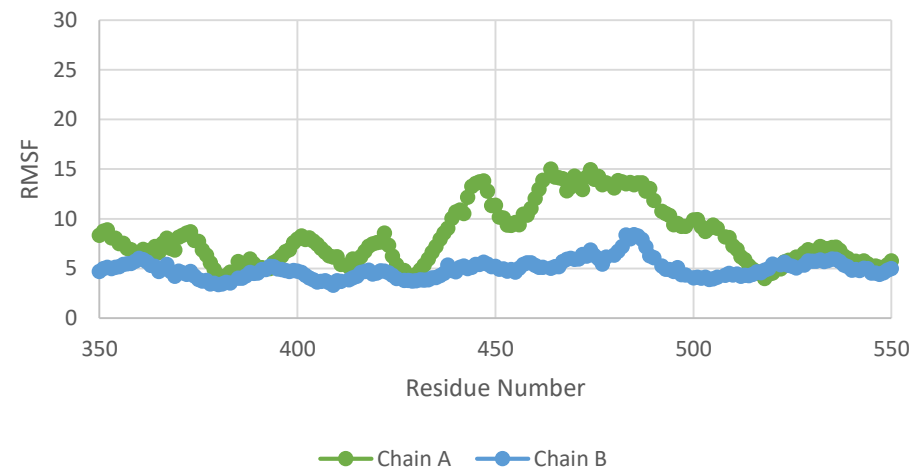

### Repeat C

RMSF WT-Trimer

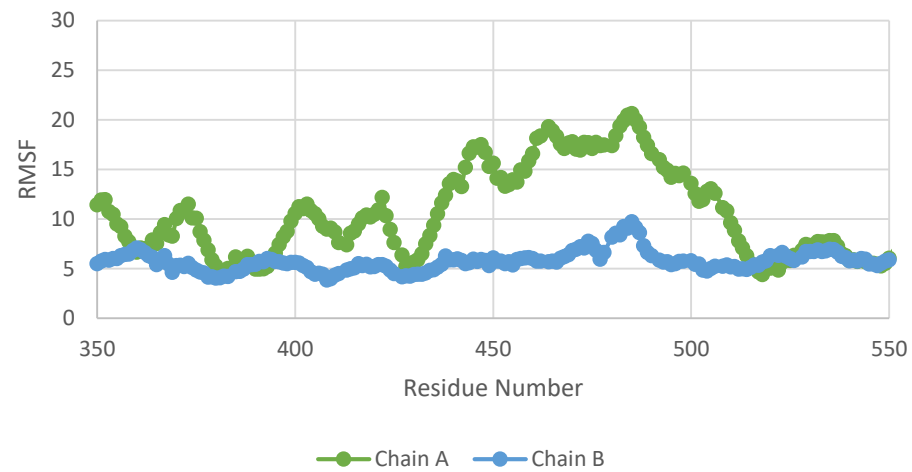

Supplement: Supplementary file 1 — Supplementary Figure S1. [file 41598_2022_20656_MOESM1_ESM.pdf]
