# Supplementary material for: Flap structure within receptor binding domain of SARS-CoV-2 spike periodically obstructs hACE2 Binding subdomain bearing similarities to HIV-1 protease flap
Source: Sci Rep. 2022 Sep 28;12:16236. doi: 10.1038/s41598-022-20656-z (PMC9517965; doi:10.1038/s41598-022-20656-z)

Repeat A

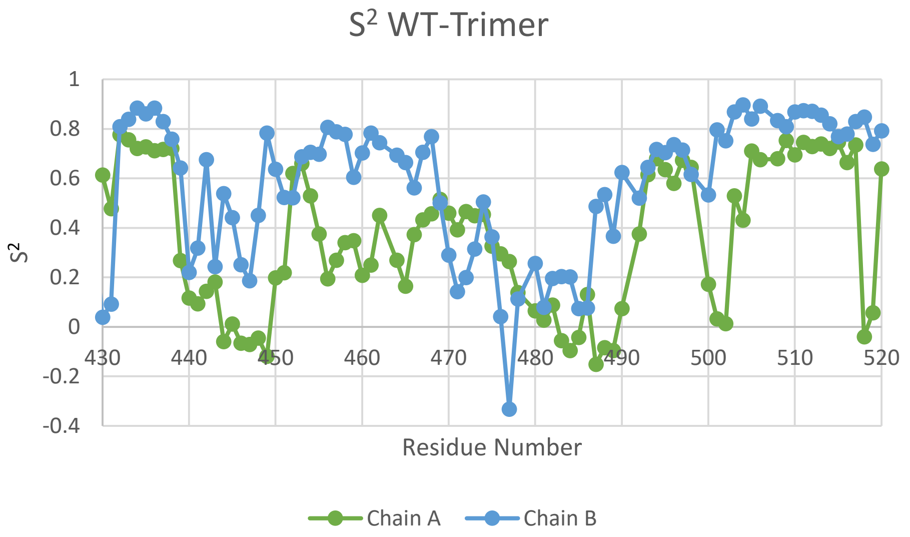

Repeat B

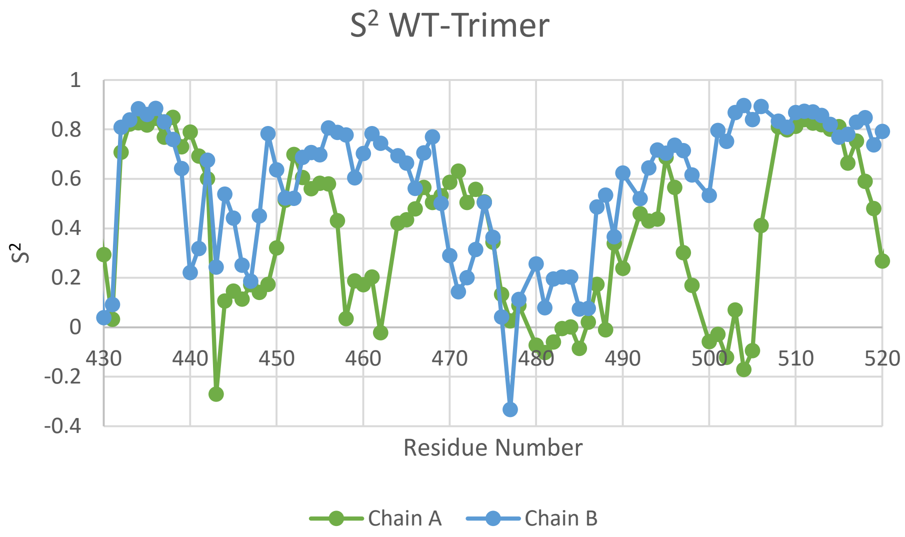

Repeat C

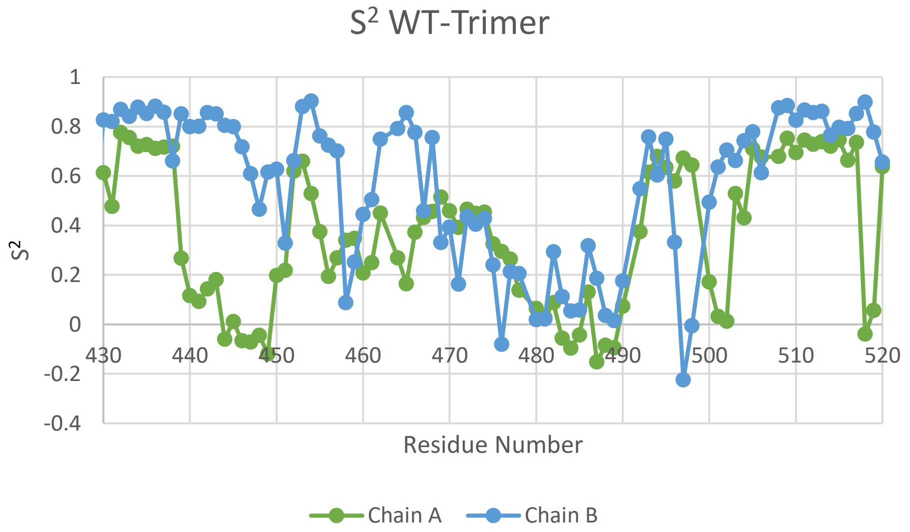

Supplement: Supplementary file 2 — Supplementary Figure S2. [file 41598_2022_20656_MOESM2_ESM.pdf]
