# Supplementary material for: Flap structure within receptor binding domain of SARS-CoV-2 spike periodically obstructs hACE2 Binding subdomain bearing similarities to HIV-1 protease flap
Source: Sci Rep. 2022 Sep 28;12:16236. doi: 10.1038/s41598-022-20656-z (PMC9517965; doi:10.1038/s41598-022-20656-z)

Fig. S3 Example of flap residue ARG466 correlation function in Up versus Down state. In the Down state, ARG466 (mostly side chain atoms) has dominant energetic interactions with its neighboring NTD residues: THR114, THR 167, GLU 132, ILE231-ILE233

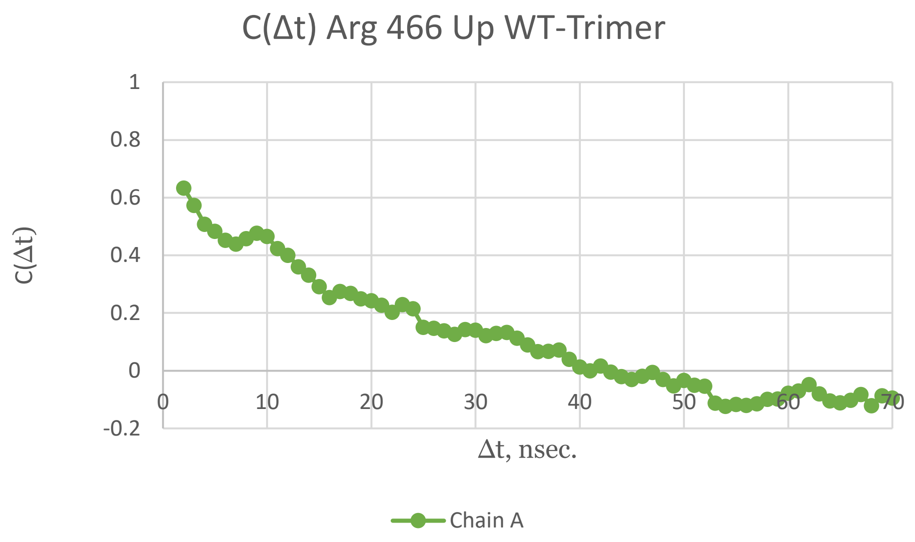

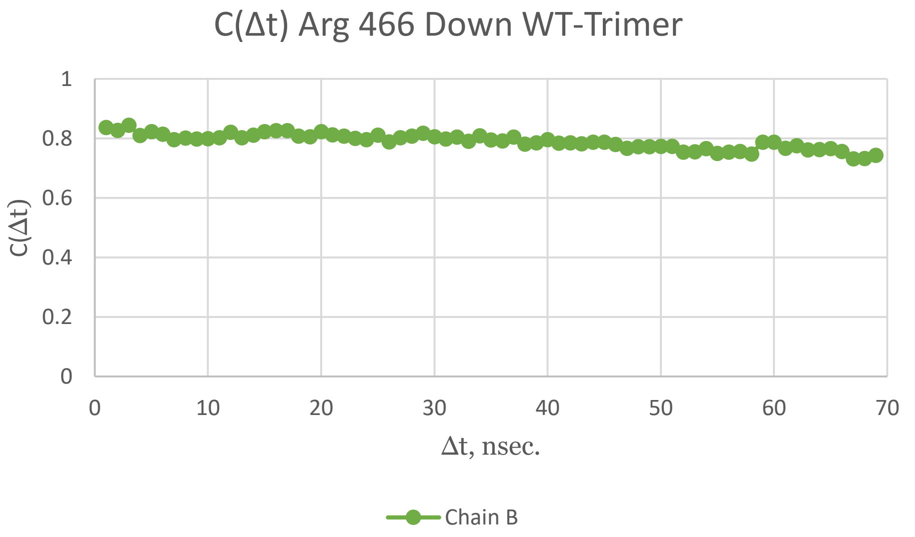

Supplement: Supplementary file 3 — Supplementary Figure S3. [file 41598_2022_20656_MOESM3_ESM.pdf]
