# Supplementary material for: Flap structure within receptor binding domain of SARS-CoV-2 spike periodically obstructs hACE2 Binding subdomain bearing similarities to HIV-1 protease flap
Source: Sci Rep. 2022 Sep 28;12:16236. doi: 10.1038/s41598-022-20656-z (PMC9517965; doi:10.1038/s41598-022-20656-z)
Supplement: Supplementary file 5 — Supplementary Table S2. [file 41598_2022_20656_MOESM5_ESM.pdf]

```
#=======================================
#
# Aligned_sequences: 2
# 1: EMBOSS_001
# 2: EMBOSS_001
# Matrix: EBLOSUM62
# Gap_penalty: 10.0
# Extend_penalty: 0.5
#
# Length: 209
# Identity:       5/209 ( 2.4%)
# Similarity:     8/209 ( 3.8%)
# Gaps:         192/209 (91.9%)
# Score: 11.5
#
#
#=======================================

EMBOSS_001         1 --------------------------CPFGEVFNATRFASVYAWNRKRIS
24
                                               ..|..:.||::      |.|... 
EMBOSS_001         1 GLEHMADEEKLPPGWEKRMSRSSGRVYYFNHITNASQ------WERPSG-
43

EMBOSS_001        25 NCVADYSVLYNSASFSTFKCYGVSPTKLNDLCFTNVYADSFVIRGDEVRQ
74

EMBOSS_001        44 --------------------------------------------------
43

EMBOSS_001        75 IAPGQTGKIADYNYKLPDDFTGCVIAWNSNNLDSKVGGNYNYLYRLFRKS
124

EMBOSS_001        44 --------------------------------------------------
43

EMBOSS_001       125 NLKPFERDISTEIYQAGSTPCNGVEGFNCYFPLQSYGFQPTNGVGYQPYR
174

EMBOSS_001        44 --------------------------------------------------
43

EMBOSS_001       175 VVVLSFELL    183

EMBOSS_001        44 ---------     43


#---------------------------------------
#---------------------------------------
```
