# Supplementary material for: Flap structure within receptor binding domain of SARS-CoV-2 spike periodically obstructs hACE2 Binding subdomain bearing similarities to HIV-1 protease flap
Source: Sci Rep. 2022 Sep 28;12:16236. doi: 10.1038/s41598-022-20656-z (PMC9517965; doi:10.1038/s41598-022-20656-z)
Supplement: Supplementary file 6 — Supplementary Table S3. [file 41598_2022_20656_MOESM6_ESM.pdf]

```
########################################
# Program: needle
# Rundate: Tue 24 May 2022 21:14:12
# Commandline: needle
#    -auto
#    -stdout
#    -asequence emboss_needle-E20220524-213020-0874-21503531-p1m.asequence
#    -bsequence emboss_needle-E20220524-213020-0874-21503531-p1m.bsequence
#    -datafile EBLOSUM62
#    -gapopen 10.0
#    -gapextend 0.5
#    -endopen 10.0
#    -endextend 0.5
#    -aformat3 pair
#    -sprotein1
#    -sprotein2
# Align_format: pair
# Report_file: stdout
########################################

#=======================================
#
# Aligned_sequences: 2
# 1: EMBOSS_001
# 2: EMBOSS_001
# Matrix: EBLOSUM62
# Gap_penalty: 10.0
# Extend_penalty: 0.5
#
# Length: 209
# Identity:      19/209 ( 9.1%)
# Similarity:    31/209 (14.8%)
# Gaps:         136/209 (65.1%)
# Score: 12.5
#
#
#=======================================

EMBOSS_001         1 CPFGEVFNATRFASVYAWNRKRISNCVADYSVLYNSASFSTFKCYGVSPT     50
                                                                       
EMBOSS_001         1 --------------------------------------------------      0

EMBOSS_001        51 KLNDLCFTNVYADSFVIRGDEVRQIAPGQTGKIADYNYKLPDDFTGCVIA    100
                                                                ..:..
EMBOSS_001         1 ---------------------------------------------PQITL      5

EMBOSS_001       101 WNSNNLDSKVGGNYNYLYRLFRKSNLKPFERDISTE--IYQAGSTP----    144
```

```
                     |....:..|:||            .||....|...:  :.:..|.|
EMBOSS_001         6 WQRPLVTIKIGG------------QLKEALLDTGADDTVLEEMSLPGRWK     43

EMBOSS_001       145 ---CNGVEGFNCYFPLQSY--------GFQPTNGVGYQPYRVVVLSFELL    183
                        ..|:.||   ..::.|        |.:....|...|..|.::...||
EMBOSS_001        44 PKMIGGIGGF---IKVRQYDQILIEICGHKAIGTVLVGPTPVNIIGRNLL     90

EMBOSS_001       184 ---------    183

EMBOSS_001        91 TQIGCTLNF     99


#---------------------------------------
#---------------------------------------
```
